# Complete Genome Sequence of Escherichia albertii Strain 1551-2, a Potential Extracellular and Intracellular Pathogen

**DOI:** 10.1128/genomeA.00075-18

**Published:** 2018-03-01

**Authors:** Fabiano Teodoro Romão, Rodrigo Tavanelli Hernandes, Tadasuke Ooka, Tetsuya Hayashi, Vanessa Sperandio, Tânia Aparecida Tardelli Gomes

**Affiliations:** a Departamento de Microbiologia, Imunologia e Parasitologia, Escola Paulista de Medicina, Universidade Federal de São Paulo, São Paulo, São Paulo, Brazil; b Department of Microbiology and Biochemistry, University of Texas Southwestern Medical Center, Dallas, Texas, USA; c Departamento de Microbiologia e Imunologia, Instituto de Biociência, Universidade Estadual Paulista Júlio de Mesquita Filho (UNESP), Botucatu, São Paulo, Brazil; d Department of Microbiology, Graduate School of Medical and Dental Sciences, Kagoshima University, Kagoshima, Japan; e Department of Bacteriology, Faculty of Medical Sciences, Kyushu University, Fukuoka, Japan

## Abstract

Escherichia albertii has recently been recognized as an emerging human and bird enteric pathogen. Here, we report the complete chromosome sequence of a clinical isolate of E. albertii strain 1551-2, which may provide information about the pathogenic potential of this new species and the mechanisms of evolution of Escherichia species.

## GENOME ANNOUNCEMENT

Diarrhea is one of the main causes of infant mortality worldwide, mainly in the developing world, and many enteropathogens are associated with this disease (1), including Escherichia albertii, which is emerging as an important human enteropathogen (2–4). E. albertii strains have similarity with enteropathogenic Escherichia coli (EPEC) strains, as they harbor a pathogenicity island called the locus of enterocyte effacement (LEE) and multiple effector proteins (5, 6). These effectors are injected into the host cell cytosol by a type III secretion system (T3SS) encoded by the LEE (7).

Here, we report the complete chromosome sequence of E. albertii strain 1551-2. This strain was previously classified as an atypical EPEC (8) strain. E. albertii 1551-2 was the sole pathogenic isolate detected in the stool from a 1-year-old child with acute diarrhea in 1989 (8). The E. albertii 1551-2 strain was previously reported to have the potential to invade enterocytes *in vitro* and *in vivo,* to persist inside these cells (9–12), and to form biofilms *in vitro* (13, 14).

A genomic DNA library from E. albertii strain 1551-2 was sequenced by a PacBio RS sequencing system (Pacific Biosciences), generating 82,693 reads (764,335,723 bp in total length). The sequence reads were assembled, protein-coding sequences were predicted, and functional annotation was performed with the PacBio software SMRT Analysis 2.0 (Pacific Biosciences). RNA genes were predicted using the Microbial Genome Annotation Pipeline (MiGAP [http://www.migap.org/index.php/en]). We performed a manual inspection for start codons and potential pseudogenes.

The complete genome sequence of E. albertii strain 1551-2 consists of a chromosome of 4,730,877 bp in size with 49.9% G+C content, and it contains 4,533 coding sequences (CDSs), 7 rRNA (*rrn*) operons, and 89 tRNA genes. No plasmids were detected. A preliminary analysis of the genome sequence confirmed the presence of the LEE genes, which includes the intimin gene (*eae*) and other virulence factor-encoding genes.

The E. albertii 1551-2 sequence could contribute to the search for new virulence markers and genes that are potentially involved in bacterial colonization, cell invasion, and intracellular persistence. Additionally, this work could contribute to the studies of genome plasticity and mechanisms that are evolutionarily shared among pathogens of the Escherichia genus and of other members of the Enterobacteriaceae family.

### Accession number(s).

The complete genome sequence of E. albertii strain 1551-2 has been deposited in GenBank under the accession no. CP025317.
